# The clinical outcomes of patients who developed typical atrial flutter on class 1C anti arrhythmic medications treated with hybrid approach

**DOI:** 10.1002/clc.23193

**Published:** 2019-05-14

**Authors:** Lior Grossman, Moshe Katz, Roy Beinart, Eyal Nof

**Affiliations:** ^1^ Leviev Heart Institute Sheba Medical Center Tel Hashomer Ramat Gan Israel; ^2^ Sackler School of Medicine Tel Aviv University Tel Aviv Israel

**Keywords:** ablation, atrial fibrillation, atrial flutter

## Abstract

**Introduction:**

A common approach to patients, who developed atrial flutter secondary to treatment with class 1C anti‐arrhythmic drugs for atrial fibrillation (AF) (1C flutter), is a hybrid approach: ablation of the Cavo‐Tricuspid isthmus (CTI) and continuation 1C medical treatment to prevent recurrence of AF. We aim to explore the clinical outcome of patients treated in this approach**.**

**Methods and Results:**

Two hundred and four consecutive patients who underwent ablation for typical AFL at a tertiary medical center between 2010 and 2016 were enrolled and followed up. The clinical outcome of patient treated by the hybrid approach (treatment group; n = 67) was compared to patient without history of AF (control group; n = 137). The primary endpoint was time to occurrence of AF. Twenty‐eight (41.8%) patients in treatment group had AF occurrence in 1 year, including 9 (13.4%) patients who needed to escalate anti‐arrhythmic drug treatment to class III, and 11 (16.4%) patients who underwent AF ablation. In comparison, only 21 (15.3%) patients in control group had occurrence during the first year after ablation. The median time from ablations till AF occur was 106 ± 481 days in treatment group, and 403 ± 668 days in control group (*P* < .01).

**Conclusions:**

There is a relatively high rate of AF recurrence in patients treated with the hybrid approach during the first year after CTI ablation. An alternative approach should be considered in this selected population.

## 1 INTRODUCTION

Class 1C anti‐arrhythmic medications are first‐line therapy for those who present with atrial fibrillation (AF) and do not have structural heart disease.^1^ The association and dissociation rate of the class 1C drugs is relatively slow comparing with other sodium blocking drugs (class Ia and Ib). Therefore, the short time for drug‐channel dissociation in high heart rate leads to increasing number of blocked channels and furthermore, to a relatively higher effect (this drug character called “use‐dependent activity”). However, the use‐dependent activity could lead to a pro‐arrhythmic arrhythmia such as cavo‐tricuspid isthmus (CTI)‐dependent atrial flutter (typical atrial flutter). This situation is classically determined as 1C flutter.^2^ When drug therapy for AF fails, an ablation that includes pulmonary veins isolation (PVI) is usually performed. The complications incidence in this procedure is relatively low. However, major complications such as tamponade and stroke can occur,^3^ and therefore many physicians opt for a common approach of exhausting medical treatment first.

Typical isthmus‐dependent atrial flutter (AFL) is an arrhythmia that is relatively easy to treat by performing ablation of the isthmus between the inferior vena cava and the tricuspid valve in the right atrium. Complications rate in this procedure are significantly lower than the complications rates of pulmonary vein isolation ablation.^3^, ^4^, ^5^

Nowadays, many physicians offer a hybrid therapy approach to patients who develop 1C flutter.^6^ This treatment includes ablation of the CTI in the right atrium for treating the AFL^7^, ^8^ and continuing the medical treatment with 1C anti‐arrhythmic drugs to hopefully continue prevent AF. In some patients, there is a recurrence of the initial arrhythmia (AF) which usually indicates anti‐arrhythmic drug treatment failure^9^, ^10^ or a decrease in the drug effectiveness. In these cases, the patient will need either an upgrade of the medical treatment for second line drugs such as amiodarone or sotalol that are known for their broad adverse drug reaction or pulmonary vein isolation (PVI) ablation. The success rate of PVI ablation for preventing symptomatic AF recurrence is approximately 50% to 80% depending on whether AF was paroxysmal or persistent, and whether an anti‐arrhythmic drug treatment was given.^11^, ^12^ An alternative strategy, suggested by others, claims that performing PVI and CTI in one procedure may be more efficient because of the high rate of AF recurrence.

The clinical outcomes of patients treated in hybrid approach is unknown.

## 2 METHODS

### 2.1 Study population

Two hundred and four consecutive patients who underwent ablation for typical AFL at a tertiary medical center between February 2010 and November 2016 were enrolled and followed up. Sixty‐seven patients (33%) with past medical history of AF who were treated with 1C antiarrhythmic drugs for AF prevention and who were without documentation of AF recurrence on treatment, were allocated to the hybrid strategy. They underwent CTI ablation and continued the medical treatment with 1C drugs (treatment group). Of those, 46 patients were treated with propafenone and 20 patients were treated with flecainide. In one patient, the data of the specific 1C drug was missing. One hundred thirty‐seven patients (67%) without past medical history of AF served as control group. Thus, these patients underwent CTI ablation only.

Patients who had both AF and AFL arrhythmic disorders at presentation were excluded from this study. The study population was collected and enrolled from the Center for Arrhythmias and Rhythms database in Sheba Medical Center, after performing search for CTI ablation for AFL. This database is a prospectively collected registry and includes all patients undergoing an ablation procedure. A total of 508 patients detected in this search, and after applying the exclusion criteria and emitting patients without any follow up data, the study population was established and allocated into the two study groups: patients with 1C flutter and patients with typical AFL with no history of AF or 1C treatment (shown in Figure S1).

### 2.2 CTI ablation procedure

All patients were diagnosed with a typical counter clock wise AFL (CTI dependent) according to a 12 lead ECG and/or during electrophysiological study. CTI ablation was performed to achieve bi‐directional conduction block across the CTI line.

Complications of the procedure were documented and divided into three categories: Vascular access complication that includes a major hematoma or necrosis at access site, tamponade and a need of permanent pacemaker.

### 2.3 Clinical follow‐up

The cohort was followed up in our institute. Every ambulatory visit, hospital admissions, and change in medical treatment was documented. In each visit comprehensive anamnesis was taken, physical examination was done, and ECG was performed. Moreover, we examined 24‐hour Holter monitoring to look for asymptomatic AF recurrence.

Missing information was completed by telephone questionnaire.

The primary endpoint was time to AF occurrence. This time was defined and determined by the shortest period between CTI ablation and one of the following:

1. Time of switching class 1C antiarrhythmic drug to class III antiarrhythmic drug.
2. Time to PVI.
3. Time of documented AF on ECG or Holter.

In addition data of mortality and procedure complication were collected and compared.

### 2.4 Statistical analysis

Normal distributed continuous variables were reported as mean ± SD, while skewed variables were reported as median and interquartile range (IQR).

Categorical variables were reported as number and percentage.

Continuous variables were compared by independent sample *t* test or by Mann‐Whitney test.

Comparison of categorical variables between groups was performed using χ² test or by using Fisher's exact test. The time to AF recurrence is graphically displayed using Kaplan‐Meier curve, censored at the time of death or date of last follow‐up. A Log‐Rank test was used to compare survival between groups.

Univariate Cox regression was used to describe the crude hazard ratio for AF occurrence.

All statistical tests were two‐tailed. *P* < .05 was considered statistically significant.

SPSS software was used for statistical analysis (IBM SPSS statistics, Version 25, IBM Corp, Armonk, New York, 2017).

This study was approved by the institutional review board (IRB).

## 3 RESULTS

### 3.1 Study population

The patients were followed for a mean time of 912 ± 766 days. Sixty‐seven (33%) patients with 1C flutter were included in treatment group and 137 (67%) in control group. Baseline patient characteristics are outlined in Table 1. In general, both patient groups were well balanced however patients in the control group had more diabetes mellitus (DM) (42% vs 19%; *P*‐value = .001), and more ischemic heart disease (IHD) (40% vs 12%; *P*‐value <.001). In addition, there were more males in the control group (82% vs 69%; *P*‐value = .025).

**Table 1:** Baseline characteristics of the study population

	Treatment group N = 67	Control group N = 137	*P*‐value
Age (years)	65.0 ± 8.9	66.3 ± 11.6	.402
Gender, male	46 (69)	113 (82)	.025
BMI (kg/cm^2^)	28.5 ± 4.7	28.8 ± 4.9	.705
Co‐morbidities			
Creatinine (mg/dL)	0.99 ± 0.2	1.14 ± 0.41	.001
Past PVI	5 (8)	0	.003
Past CTI ablation	5 (8)	13 (10)	.632
HTN	32 (48)	78 (57)	.217
Valvular surgery	6 (9)	15 (11)	.660
Severe valvular disease	1 (2)	4 (3)	1
DM	13 (19)	58 (42)	.001
CVA	7 (10)	17 (12)	.683
IHD	8 (12)	55 (40)	<0.001
Echocardiography			
LVEF %	60 [55‐60]	55 [40‐60]	.001
LVEF < 50	2 (3)	47 (35)	<0.001
LAD (cm)	4.1 ± 0.6	4.4 ± 0.5	.048
LAA (cm^2^)	23.0 ± 5.1	24.6 ± 4.5	.091
AFL duration:			.8
Less than 1 month	11 (16)	20 (15)	
One month to 1 year	47 (70)	94 (69)	
More than 1 year	9 (13)	23 (17)	
Drugs			
BB	41 (62)	80 (58)	0.612
CCB	3 (5)	3 (2)	0.393

Abbreviations: BB, beta blockers; BMI, body mass index; CCB, calcium channel blockers; CTI, cavo‐tricuspid isthmus; CVA, cerebrovascular accident; DM, diabetes mellitus; HTN, hypertension; IHD, ischemic heart disease; LAA, left atrium area; LAD, left atrium diameter; LVEF, left ventricular ejection fraction; PVI, pulmonary vein isolation.

Categorical variable presented as number (%) continues variable presented as mean ± SD for normal distributed variables or as median [IQR] for non‐normal distributed variable.

Echocardiographic parameters show that in the control group more patients had reduced left ventricular ejection fraction (LVEF) (35% vs 3%; *P*‐value <.001), and also a larger mean left atrium diameter (LAD) (4.4 ± 0.5 vs 4.1 ± 0.6 cm; *P*‐value = .048).

### 3.2 AF occurrence

**Figure 1:**
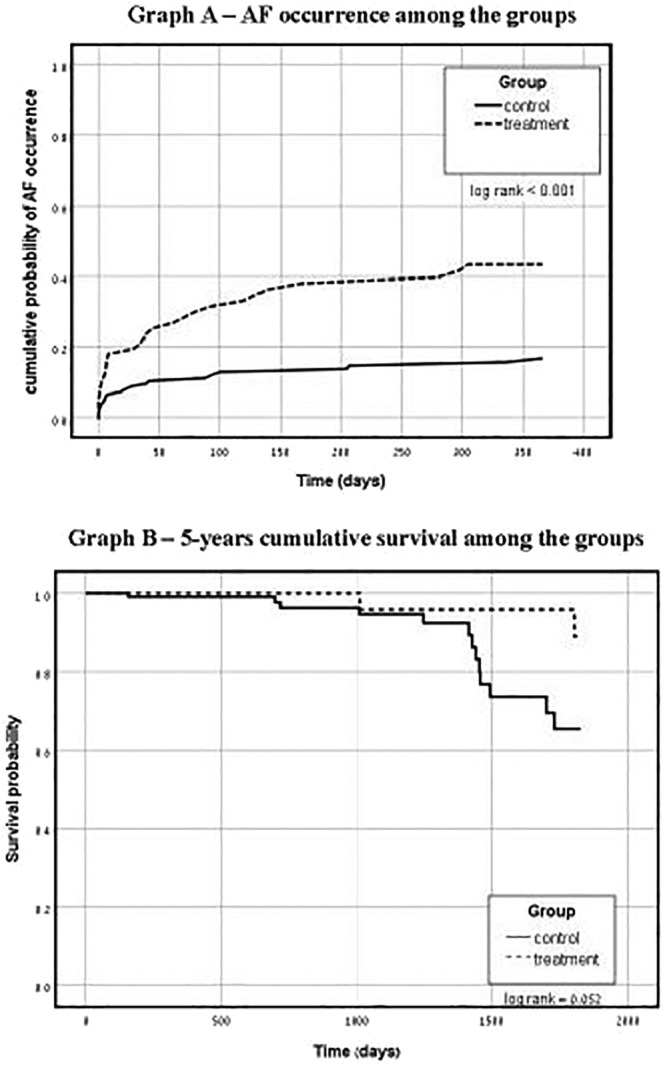
A, Kaplan‐Meier (One‐minus survival) Plot displaying time to occurrence—the proportion of the population with AF occurrence in 1 year (*y*‐axis) vs time (*x*‐axis). B, Kaplan‐Meier Plot displaying cumulative survival—the proportion of 5 years survival among the groups (*y*‐axis) vs time (*x*‐axis)

The cumulative probability of AF occurrence in 1 year after CTI ablation was higher in the treatment group (41.8%) vs the control group (15.3%; log‐rank *P* < .001) (Figure 1A). In the treatment group, conversion of 1C antiarrhythmic drug to class III antiarrhythmic drug was done in nine subjects (13%) and another 11 subjects (16%) underwent PVI during the first year of follow‐up. In control group, only 21 (15.3%) patients had AF occurrence during the first year of follow‐up. The median follow‐up time for the entire cohort was 678 days. The median time to AF occurrence during all study follow‐up was 106 ± 481 days in the treatment group and 403 ± 668 days in the control group.

Moreover, in the treatment group, we performed subgroup analysis of those who underwent or did not undergo previous PVI. Five patients were with history of PVI ablation. We compared their AF occurrence with the rest of the 62 patients in the treatment group who did not undergo PVI ablation. The baseline characteristics of those subgroups is shown in Table S1. There was a tendency towards lower rates of AF recurrence in those that had previous PVI ablation, but this did not reach statistical significance probably due to small number of patients in each group. Only one patient had AF occurrence (20%) in the previous PVI subgroup, in comparison to 27 patients (44%) who had AF occurrence in the second subgroup (Figure S2).

### 3.3 Predictors of AF occurrence

Baseline characteristics were evaluated as predictors for AF occurrence (Table 2). In univariate Cox regression analysis, none of the characteristics were found statistically significant as predictors for AF occurrence.

**Table 2:** Risk factors for AF occurrence

	HR	95% CI	*P*‐value
Age	0.996	0.971‐1.021	.752
Gender	0.843	0.421‐1.690	.631
BMI	1.000	0.943‐1.061	.996
Creatinine	0.808	0.333‐1.960	.638
History of PVI	1.326	0.183‐9.607	.78
DM	1.690	0.881‐3.241	.114
IHD	1.604	0.8193‐3.138	.168
LVEF	1.006	0.978‐1.034	.681
LVEF < 50	1.444	0.699‐2.980	.321
LAD	0.807	0.408‐1.596	.538

Abbreviations: BMI, body mass index; CI, confidence interval; HR, hazards ratio; DM, diabetes mellitus; IHD, ischemic heart disease; LAD, left atrium diameter; LVEF, left ventricular ejection fraction; PVI, pulmonary vein isolation.

However, multivariate analysis comparing the 1‐year occurrence of AF between the study groups, standardized for body mass index; diabetes mellitus; ischemic heart disease; LAD; left ventricular ejection fraction; PVI, found that the risk for AF occurrence in 1 year in treatment group is higher than in control group (hazards ratio [HR] 3.708, 95% confidence interval [CI] 1.625‐8.460; *P* = .002) (Table 3).

**Table 3:** multivariate analysis for AF occurrence

	HR	95% CI	*P*‐value
1C flutter	3.708	1.625‐8.460	.002
Age	1.003	0.963‐1.045	.869
Gender	2.287	0.828‐6.315	.11
LAD	0.926	0.460‐1.865	.83

Abbreviations: CI, confidence interval; HR, hazards ratio; LAD, left atrium diameter.

Adjusted for body mass index; diabetes mellitus; ischemic heart disease; left atrium diameter; left ventricular ejection fraction; pulmonary vein isolation.

### 3.4 CTI ablation complication

The CTI ablation procedural complication incidence was low in both groups (Table 4).

**Table 4:** CTI ablation complications

	Treatment group N = 67	Control group N = 137	All N = 204
Total complication	1 (1.5%)	3 (2.2%)	4 (2%)
Vascular access	0	2 (1.5%)	2 (1%)
Tamponade	0	1 (0.7%)	1 (0.5%)
Need for permanent pacemaker	1 (1.5%)	0	1 (0.5%)

Abbreviations: AF, atrial fibrillation; CTI, cavo‐tricuspid isthmus.

Vascular access includes complication of major hematoma or necrosis at access site; Tamponade refers to right side tamponade.

### 3.5 Mortality

During the first 5 years of follow‐up, 15 (7.4%) patients died. Thirteen patients died in control group and two patients died in treatment group. Ten patients died after more than 3.5 years of follow‐up. The all‐cause mortality rate was not significantly different between the groups. The results for long‐term follow‐up are displayed in a Kaplan‐Meier survival plot (Figure 1B).

## 4 DISCUSSION

In this study, we aimed to examine AF occurrence rate in patients who present with 1C flutter and are treated with hybrid therapy approach.

The main finding in this study is the high occurrence rate of AF after CTI ablation in patients with 1C flutter. The results of this study show that hybrid therapy approach for 1C flutter fails to achieve long lasting free AF survival in the majority of the patients, in a relatively short period of time after CTI ablation. Over 40% of patients treated with hybrid therapy had AF recurrence in 1 year. No demographic factor, comorbidity, echocardiographic factor or medical treatment, was found to be a predictor for AF occurrence in this study. The only variable that was found to increase the risk for shorter AF occurrence was the fact of having 1C flutter with history of AF.

In order to strengthen our conclusions, we also compared this rate to the AF occurrence rate of typical flutter patients without AF history undergoing CTI ablation. This comparison was made to show that CTI ablation is effective in arrhythmia management control in those with AFL only but less effective as sole therapy in those with history of AF.

The result of our study strongly suggests that hybrid therapy approach for 1C flutter do not show results that are as good as the results for CTI ablation as monotherapy for patients with typical AFL suggesting that some patients presenting with 1C flutter and history of AF should undergone a combined procedure of CTI ablation together with PVI at time presentation. Once they develop AFL, 1C therapy with also fail in preventing AFIB. Moreover, our data suggests patients with only typical AFL without history of AFIB, should undergo only CTI ablation. This corresponds to previous data.^13^

In previous study, a large LAD was found to be a predictor of AF occurrence.^14^, ^15^, ^16^, ^17^ This was not found to be a predictor in our study. Most probably because of the fact that the LAD was not large in both of the study groups. Other predictors include presence of structural heart disease,^15^ history of AF episode before ablation^5^, ^17^, ^18^, ^19^ and failure of multiple anti arrhythmic medications.^15^ None of those were predictive in our selected patient population.

Next, when we divided the group of 1C flutter to patients with history of PVI and patients without history of PVI, although not statistically significant, there seems to be a tendency towards a lower rate of AF recurrence during first year of follow‐up among patients with history of PVI, and we believe that those patients may benefit more from the hybrid therapy approach and have a better outcome. These groups were not statistically different because the study was underpowered for this comparison. Only five patients had history of PVI. PVI ablation allows longer time of freedom from AF. Thus, patients with 1C flutter that already had an ablation for AF in the past were more “protected” than patients that did not have an ablation. This protection is reflected in the lower rate of AF recurrence.

The higher mortality rate that was found in the control group, is not statistically significant and even if reflects a tendency, it could be explained by the fact that patients in this group had more co‐morbidities at baseline, and as a consequence were prone to disabilities, clinical deterioration, and death.

The total complication rate of the CTI ablation procedure in this study is compatible with the literature.^3^, ^4^, ^5^

Looking for the future, we believe that a randomized prospective study should be performed to compare a combination procedure of CTI and PVI ablation in patients with history of AF presenting now with 1C flutter to CTI ablation only.

### 4.1 Limitations

This study is a retrospective cohort single center study with relatively small number of participants. However, being a single center study ensures that data acquisition, ablation procedure and follow up were uniform. Moreover, the small sample size resulted in underpowered subgroup analysis. Due to the retrospective, observational nature of the study we had to complete the missing information by telephone questionnaire, which may result in recall bias.

## 5 CONCLUSIONS

The strategy of CTI ablation and continuing medical therapy with class 1C antiarrhythmic drugs after ablation (hybrid approach) results in high rate of AF recurrence in relatively short period. Hence, different strategy, such as combined CTI and PVI ablation should be considered. However, the superiority of one strategy over another should be tested in randomized clinical trial.

## Supporting information


**FIGURE S1** Study population

**FIGURE S2** Atrial fibrillation (AF) occurrence among patients with and without history of PVI

**TABLE S1** Baseline characteristics of subgroups
