# Supplementary figures and images for: The clinical outcomes of patients who developed typical atrial flutter on class 1C anti arrhythmic medications treated with hybrid approach
Source: Clin Cardiol. 2019 May 14;42(7):678–83. doi: 10.1002/clc.23193 (PMC6605003; doi:10.1002/clc.23193)

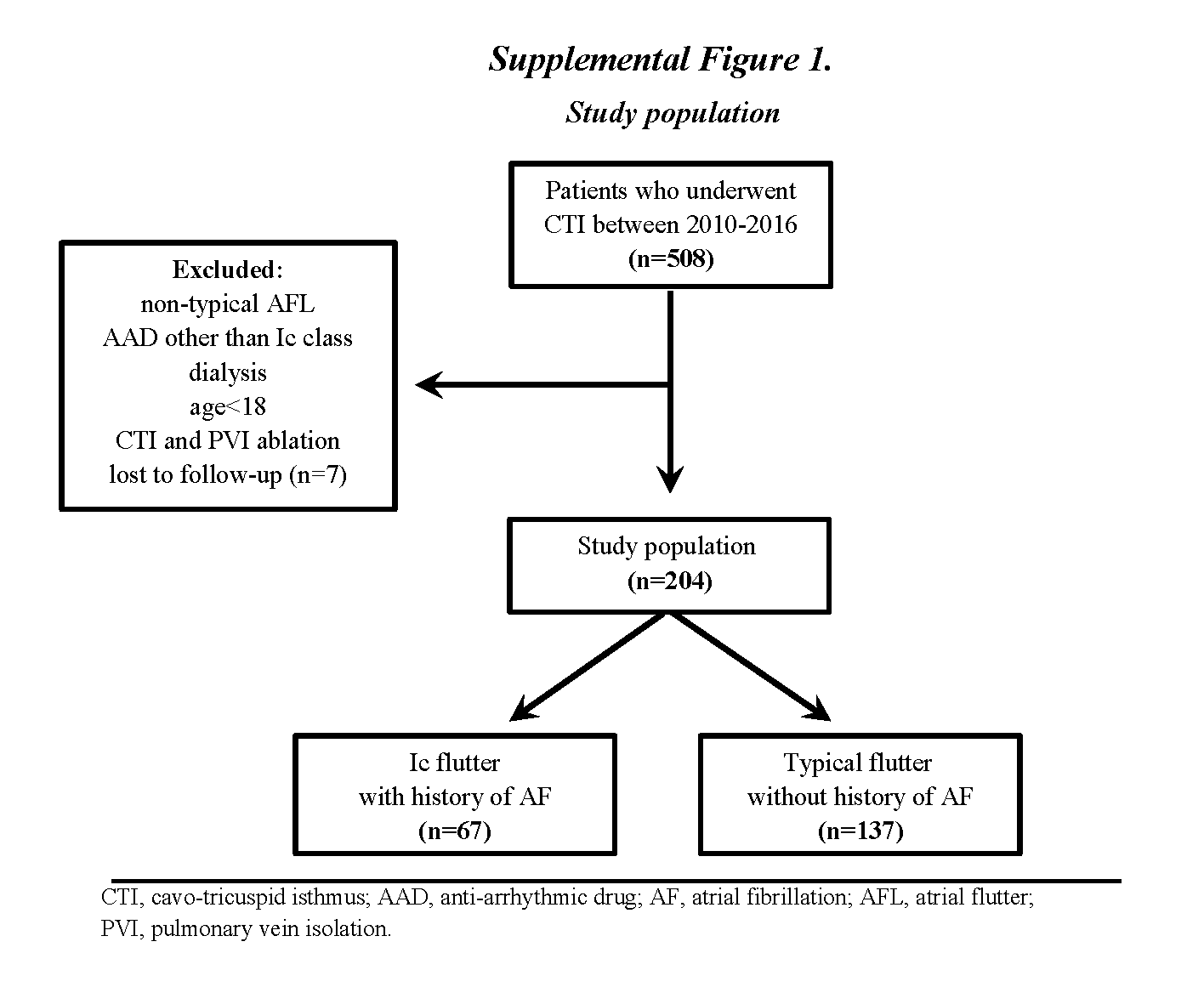

Supplement: Supplementary file 1 — FIGURE S1 Study population [file CLC-42-678-s001.tiff]

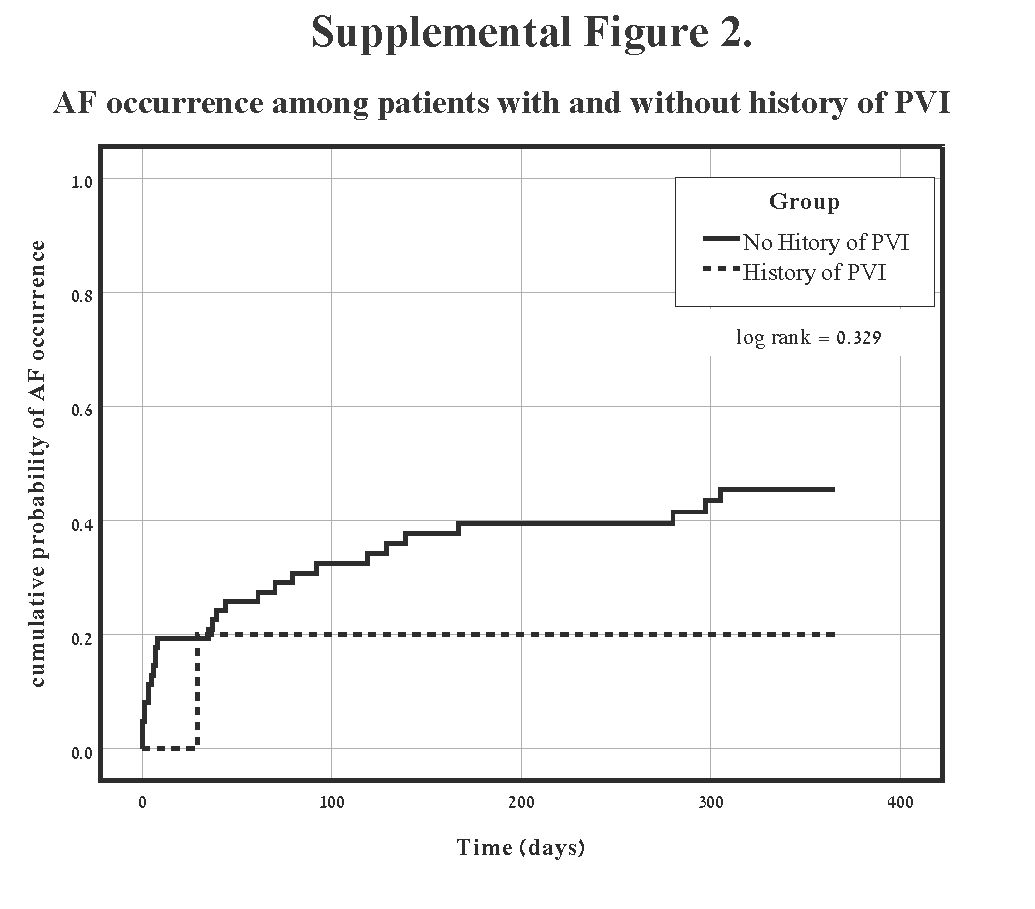

Supplement: Supplementary file 2 — FIGURE S2 Atrial fibrillation (AF) occurrence among patients with and without history of PVI [file CLC-42-678-s002.tiff]
