# Supplementary material for: The clinical outcomes of patients who developed typical atrial flutter on class 1C anti arrhythmic medications treated with hybrid approach
Source: Clin Cardiol. 2019 May 14;42(7):678–83. doi: 10.1002/clc.23193 (PMC6605003; doi:10.1002/clc.23193)
Supplement: Supplementary file 3 — TABLE S1 Baseline characteristics of subgroups [file CLC-42-678-s003.docx]

| ***Supplemental Table 1.***  ***Baseline characteristics of subgroups*** | | | |  |  |
| --- | --- | --- | --- | --- | --- |
|  | **Previous PVI**  **N=5** | **No Previous PVI**  **N=62** | **p-value** | | |
| **Age (years)** | 61.8±8.0 | 65.2±9.0 | 0.4 | | |
| **Gender, Male** | 3 (60) | 43 (69) | 0.65 | | |
| **BMI (Kg/cm^2^)** | 30.9±5.6 | 28.6±4.6 | 0.24 | | |
| **Co-morbidities** | | | | | |
| **Creatinine (mg/dL)** | 0.97±0.2 | 1.00±2.0 | 0.76 | | |
| **Past CTI Ablation** | 0 | 5 (8) | 1 | | |
| **HTN** | 3 (60) | 29 (47) | 0.66 | | |
| **Valvular Surgery** | 0 | 6 (10) | 1 | | |
| **Severe Valvular Disease** | 0 | 1 (2) | 1 | | |
| **DM** | 1 (20) | 12 (19) | 1 | | |
| **CVA** | 2 (40) | 5 (8) | 0.08 | | |
| **IHD** | 0 | 8 (13) | 1 | | |
| **Echocardiography** | | | | | |
| **LVEF<50** | 0 | 2 (3) | 1 | | |
| **LAD (cm)** | 4.4±1.2 | 4.1±0.5 | 0.48 | | |
| **LAA (cm^2^)** | 22.3±5.2 | 23.1±5.1 | 0.8 | | |
| **AFL Duration:** |  |  | 0.27 | | |
| **Less than one month** | 2 (40) | 9 (15) |  | | |
| **One month to one year** | 3 (60) | 44 (71) |  | | |
| **More than one year** | 0 | 9 (15) |  | | |
|  | **Drugs** |  |  | | |
| **BB** | 2 (40) | 39 (64) | 0.36 | | |
| **CCB** | 1 (20) | 2 (3) | 0.21 | | |
| Categorical variable presented as number (%) Continues variable presented as mean ± SD for normal distributed variables. BB, beta blockers; BMI, body mass index; CCB, calcium channel blockers; CTI, cavo-tricuspid isthmus; CVA, cerebrovascular accident; DM, diabetes mellitus; HTN, hypertension; IHD, ischemic heart disease; LAA, left atrium area; LAD, left atrium diameter; LVEF, left ventricular ejection fraction; PVI, pulmonary vein isolation. | | | | |  |
